# HLX in AML: novel prognostic and therapeutic target

**DOI:** 10.18632/oncotarget.705

**Published:** 2012-10-11

**Authors:** Ashley Pandolfi, Ulrich Steidl

**Affiliations:** Department of Cell Biology, Albert Einstein Cancer Center, and Ruth L. and David S. Gottesman Institute for Stem Cell Research and Regenerative MedicineBronx, NY, USA; Department of Medicine (Oncology), Albert Einstein College of Medicine, Bronx, NY, USA

Acute myeloid leukemia (AML) is an aggressive hematological malignancy with poor clinical outcome in most subtypes of the disease. Less than one third of patients achieve durable remission with current treatment regimens, and prognostication and risk stratification are challenging. We recently reported that a new non-clustered homeobox gene, H2.0-like homeobox (HLX), regulates early hematopoiesis and promotes AML in mice and humans [1]. *Hlx* was first detected via is expression in hematopoietic cells of the myelomonocytic and B lymphocyte lineages and further studies indicated a potential role for *Hlx* in hematopoietic differentiation [2-4]. We now report that HLX is elevated in the majority of AML patients, and that higher *HLX* expression correlates with poor overall survival. These findings, combined with the observation that HLX knockdown results in an inhibition of growth and clonogenicity of leukemia cells, suggest that HLX may be a novel promising prognostic and therapeutic target in AML.

Recent experimental evidence has shown that acute myeloid leukemia (AML) arises from transformed immature hematopoietic cells following the accumulation of multiple stepwise genetic and epigenetic changes in hematopoietic stem cells (HSC) and progenitors [5]. In order to identify genes involved early on in leukemogenesis, we analyzed gene expression changes in sorted hematopoietic stem and progenitor cells (HSPC) isolated during the pre-leukemic stage from bone marrow of animals of the PU.1 URE(upstream regulatory element)^Δ/Δ^ model of AML [6]. Using this strategy, we found that pre-leukemic HSPC have 4-fold elevation of *Hlx* expression, suggesting that *Hlx* overexpression is an early event in leukemogenesis and *Hlx* is involved in malignant transformation [1].

To assess the effects of HLX overexpression in wild-type HSPC *in vivo* we utilized a competitive, congenic transplantation model. Experimental elevation of HLX in HSC resulted in near complete depletion of early HSPC populations (LT-HSC, ST-HSC, MPP) and 16-fold enrichment of myeloid progenitors with a surface phenotype slightly past the GMP stage (CD45^+^Kit^−^CD34^−^CD44^high^CD49b^high^CD11b^mid^). Overexpression of HLX in HSPC *in vitro* led to a myeloid differentiation block and to formation of phenotypically similar, aberrant myeloid progenitors with unlimited serial clonogenicity. These results indicate that HLX suppresses the function of normal immature HSC and progenitors but leads to a differentiation block and confers unlimited clonogenic capacity at the level of phenotypically more mature progenitors. Because the loss of HSC did not seem to be mediated by induction of apoptosis or necrosis, we speculate that HLX exerts this dual role by triggering initial differentiation of HSC followed by suppression of terminal differentiation at a more committed progenitor level. Genetic *in vivo* modeling using an inducible system will be required to definitively clarify the role of HLX elevation at different stages of hematopoietic stem and progenitor cell differentiation *in vivo*.

Of note, although overexpression of HLX leads to the formation of immortalized myeloid progenitors with unlimited serial clonogenicity, we so far have not observed development of overt leukemia upon transplantation, suggesting that HLX elevation alone is not sufficient for full transformation. Like other homeobox genes, *Hlx* may function in concert with cofactors that could confer cell type specificity to the effects of HLX overexpression and contribute to leukemic transformation. Combinatorial genetic modeling with other disease alleles in AML will be instrumental to uncover the exact role of *Hlx* in AML pathogenesis.

Several HOX genes are expressed at high levels in subtypes of AML. Important roles in leukemic transformation have been demonstrated for specific members of the HOX-A and HOX-B cluster, and the nonclustered homeobox gene CDX2 was recently reported to be implicated in leukemogenesis [7-10]. However, the clinical significance of many known HOX genes, with the notable exception of HOXA9 [11], is largely unclear. We found that HLX overexpression is both frequent and prognostic in human AML patients. *HLX* is significantly overexpressed (2 to 16 fold) in more than 80% of patients with AML, and this occurs across all major disease subtypes and is independent of blast counts. Higher levels of HLX are associated with poor overall survival in 3 different, large cohorts of AML patients (total N=601, p=2.3×10^−6^). Importantly, the prognostic value of HLX holds up in a multivariate analysis, indicating HLX overexpression and prognostic value is an independent phenomenon across several molecular subsets of patients. Notably, high HLX expression status seems to overrule several mutations which have been associated with favorable prognosis. Patients with CEBPA or NPM1 mutations for instance only have favorable overall survival if HLX is low, but a poor prognosis if HLX is expressed at high levels. While this needs to be confirmed in independent and, ideally, prospective studies, these observations suggest broad importance of HLX for the disease biology of AML, and a role for determining HLX expression levels in the prognostication of AML patients.

Based on our findings of the significance of *HLX* overexpression in both pre-leukemic and bulk AML cells, we studied the effects of HLX reduction in both murine and human AML cells. ShRNA-mediated reduction of HLX significantly inhibits leukemic growth and clonogenic capacity, and overcomes the differentiation block of AML cells. We found that HLX regulates a set of genes which likely mediate its leukemia-promoting functions, including candidates *BTG1* (B-cell translocation gene 1) and *PAK1* (p21 protein-activated kinase 1). Intersecting the gene expression data from our *in vitro* overexpression and inhibition studies with data from genome-wide expression analysis of a large cohort of human AML patients, we identified an HLX-dependent molecular signature of genes, which again included PAK1, with prognostic value for AML patients.

In summary, our studies have identified HLX as a novel key transcription factor involved in the regulation of early hematopoiesis and AML pathogenesis, and suggest HLX as a promising new therapeutic target in AML. Additional mechanistic studies will be required to identify the transcriptional targets of HLX in HSPC and to define its functionally critical downstream pathways, which will improve our understanding of the precise role of HLX in HSPC biology and malignant transformation, and may offer additional opportunities for targeted therapeutic intervention.
